# Bile Acids and Liver Cancer: Molecular Mechanism and Therapeutic Prospects

**DOI:** 10.3390/ph17091142

**Published:** 2024-08-30

**Authors:** Xuemei Zhang, Lei Shi, Xiaona Lu, Wenlan Zheng, Jia Shi, Shihan Yu, Hai Feng, Zhuo Yu

**Affiliations:** 1 Department of Hepatopathy, Shuguang Hospital Affiliated to Shanghai University of Traditional Chinese Medicine, Shanghai 201203, China; xmzhangc@hotmail.com (X.Z.); 0012021105@shutcm.edu.cn (X.L.); zhengwenlan97@gmail.com (W.Z.); gdjscrhhhhy@gmail.com (J.S.); umbrella199809@gmail.com (S.Y.); 2 Department of Clinical Laboratory, Shuguang Hospital Affiliated to Shanghai University of Chinese Traditional Medicine, Shanghai 201203, China; sg2800@shutcm.edu.cn; 3 Institute of Infectious Disease, Shuguang Hospital Affiliated to Shanghai University of Traditional Chinese Medicine, Shanghai 201203, China

**Keywords:** bile acids, hepatocellular carcinoma, gut microbiota, inflammation, tumor immunity

## Abstract

Hepatocellular carcinoma (HCC) is a highly aggressive liver malignancy and one of the most lethal cancers globally, with limited effective therapeutic options. Bile acids (BAs), as primary metabolites of hepatic cholesterol, undergo enterohepatic circulation involving secretion into the intestine and reabsorption into the liver, and their composition is modulated in this process. Recent clinical observations have revealed a correlation between alteration in the BAs profile and HCC incidence, and the effect of various species of BAs on HCC development has been investigated. The regulatory effect of different BA species on cell proliferation, migration, and apoptosis in tumor cells, as well as their interaction with gut microbiota, inflammation, and immunity have been identified to be involved in HCC progression. In this review, we summarize the current understanding of the diverse functions of BAs in HCC pathogenesis and therapy, from elucidating the fundamental mechanisms underlying both tumor-promoting and tumor-suppressive consequences of various BA species to exploring potential strategies for leveraging BAs for HCC therapy. We also discuss ongoing efforts to target specific BA species in HCC treatment while highlighting new frontiers in BA biology that may inspire further exploration regarding their connection to HCC.

## 1. Introduction

Liver cancer is a highly lethal malignancy and ranks as one of the deadliest cancers globally, while hepatocellular carcinoma (HCC) is the most common kind of liver cancer, accounting for up to 80% of the cases [1]. The main risk factors include hepatitis B and C virus infections, excessive alcohol consumption, nonalcoholic fatty liver disease (NAFLD), and steatohepatitis [2]. Despite recent significant advancements in HCC therapeutic options, the overall prognosis is still dismal, in part because HCC pathogenesis has not been systemically and thoroughly studied [3,4]. To this end, it is still urgent to deeply dig up the key factors and the regulatory mechanism in the development of HCC, thus finding and identifying potential targets in the therapy of HCC.

Bile acids (BAs) are the primary byproduct of cholesterol metabolism in the liver that participate in the regulation of lipid, glucose, and energy metabolism [5,6]. In addition to the physiological activities, BAs act as signaling molecules to exert pathological function in a variety of diseases. Recently, the role of BAs in cancer has attracted widespread attention, and the correlation of the disorder of BA composition with HCC incidence has been evidenced in several clinical practices [5,7,8,9]. The regulation of BAs on signaling pathways in tumor cells and their interaction with gut microbiota, inflammation, and immunity have been widely investigated in HCC development [10,11,12]. Therefore, systemic elucidation of the role of BAs in HCC progression is of great significance for improving the pathogenesis and therapeutic strategies for HCC.

In the present review, we provide an overview of the research that has been conducted on the role of BAs in HCC thus far and discuss the potential approaches of treating this serious global health problem by targeting BA-based signaling.

## 2. The Regulation of BA Metabolism 

BAs are the blend of metabolic products of cholesterol, mainly consisting of primary bile acids (PBAs) and secondary bile acids (SBAs), which are produced in the liver and converted in the colon [13,14,15]. Food-derived cholesterol is digested and transported into the liver, where it is catalyzed by the family of cytochrome P450 enzymes (CYPs) to the free PBAs, mainly including cholic acid (CA) and chenodeoxycholic acid (CDCA) [16]. Most CA and CDCA are further conjugated with glycine (G) or taurine (T) to form the conjugated BAs (CBAs) before being actively secreted into the biliary canaliculus with the facilitation of the membrane transporter bile salt export pump (BSEP) and multidrug resistance-associated protein 2 (MRP2) [17,18,19]. The newly synthesized PBAs are temporarily stored in the gallbladder and released into the intestine when receiving the stimulative signals such as fat in food after eating [20,21]. In the ileum, PBAs actively transfer through the enterocytes from the luminal membrane to the basolateral membrane, with the aid of the apical sodium-dependent bile salt transporter (ASBT), the ileal BA binding protein (I-BABP), the heterodimer organic solute transporter α/β (OSTα/β), and the multidrug resistance-associated protein 2/3 (MRP2/3) [22,23]. Subsequently, PBAs return to the liver along the portal vein and are reabsorbed into the hepatocytes facilitated by sodium-taurocholate co-transporting polypeptide (NTCP) and organic anion-transporting polypeptide (OATP), completing the enterohepatic circulation [24]. Non-transported PBAs enter the colon, where they are converted by gut microbiota into the SBAs, such as deoxycholic acid (DCA), lithocholic acid (LCA), and ursodeoxycholic acid (UDCA). Part of SBAs are passively transferred into the portal vein and reabsorbed into the hepatocytes, while what is left is excreted with feces [25].

**Figure 1:**
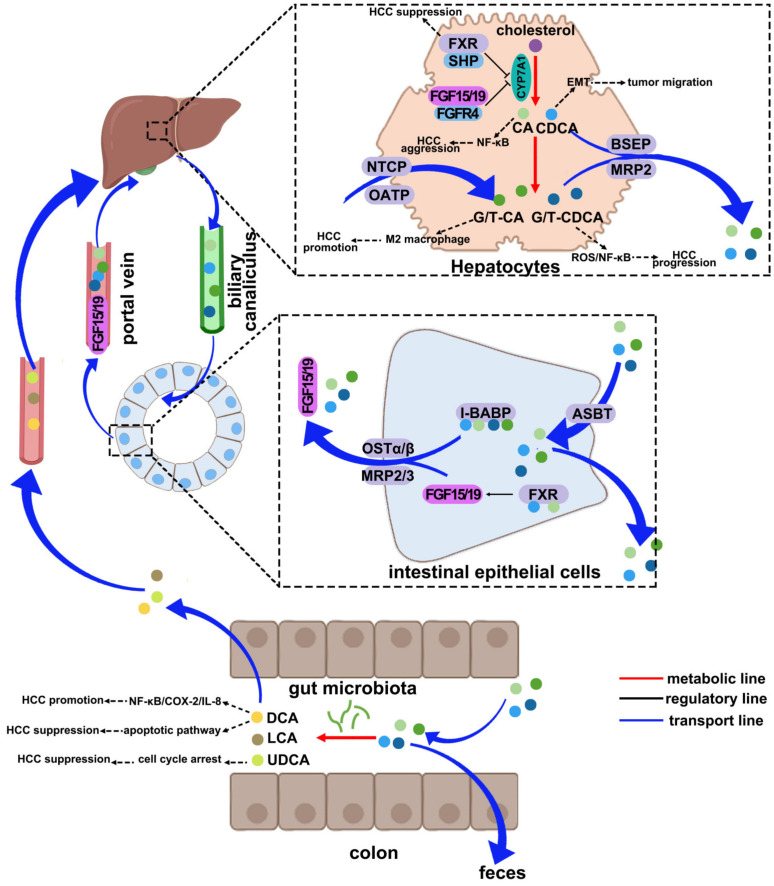
Regulation of BA metabolism and pathogenesis. Cholesterol is catalyzed to decompose free PBAs, including CA and CDCA, which were conjugated to CBAs. The majority of PBAs, both free BAs and CBAs, are excreted into the intestine, reabsorbed in the distal ileum through the active transportation, and returned to the liver via the portal vein. About 5% of PBAs enter into the colon where they are mainly transformed into SBAs by the gut microbiota, including DCA, LCA, and UDCA, followed by reabsorption through passive transportation or excretion into feces. Hepatic BSEP, MRP2, NTCP, OATP, and intestinal ASBT, I-BABP, OST-α/β, and MRP2/3 provide alternative aid to BA’s transport into the systemic circulation. In this process, FXR in the liver and intestine is essential for the balance of BA level through negative feedback regulation. Dysregulation of BA metabolism, including BA accumulation, BA imbalance, and malfunction of BA receptors, causes the pathogenesis that is shown in the figure. PBA, primary bile acid; CA, cholic acid; CDCA, chenodeoxycholic acid; CBA, conjugated bile acid; SBA, secondary bile acid; DCA, deoxycholic acid; LCA, lithocholic acid; UDCA, ursodeoxycholic acid; BSEP, bile salt export pump; MRP2, multidrug resistance-associated protein 2; NTCP, sodium-taurocholate co-transporting polypeptide; OATP, organic anion-transporting polypeptide; ASBT, apical sodium-dependent bile salt transporter; I-BABP, ileal BA binding protein; OST-α/β, organic solute transporter α/β; MRP2/3, multidrug resistance-associated protein 2/3; FXR, farnesoid X receptor; CYP7A1, cholesterol 7α-hydroxylase; SHP, small heterodimer partner; FGF15/19, fibroblast growth factor 15/19; FGFR4, fibroblast growth factor receptor 4.

The synthesis of BAs is tightly regulated to ensure proper physiological function. Cholesterol 7α-hydroxylase (CYP7A1), a member of CYPs family, acts as the rate-limiting enzyme in BA synthesis, which plays a crucial role in maintaining the balance of BAs through negative feedback [26,27]. Farnesoid X receptor (FXR), expressed in hepatocytes, functions as a negative regulator of CYP7A1. By binding to the small heterodimer partner (SHP), FXR can downregulate the expression of CYP7A1, thereby reducing the conversion of cholesterol to BAs [28]. Furthermore, FXR is also present in intestinal epithelial cells, where it can bind to BAs released into the intestine. This interaction triggers the transcription of fibroblast growth factor 15/19 (FGF15/19), a gut factor that is secreted into the portal vein [29,30]. FGF15/19 is then transported to the liver through enterohepatic circulation, where it binds to fibroblast growth factor receptor 4 (FGFR4). This binding leads to the suppression of CYP7A1 expression, ultimately inhibiting BA synthesis [31,32]. The direct and indirect negative feedback loops involving FXR and CYP7A1 ensure precise regulation of BAs synthesis, maintaining levels necessary for various physiological functions (Figure 1).

## 3. The Mechanism of BAs to Regulate HCC Development

In addition to exerting physiological functions, BAs are also involved in multiple pathological processes. Increasing evidence has established a correlation between the disorder of BA synthesis and multiple diseases including cancers [33,34]. Clinical cohort studies have revealed that abnormal changes in BA composition are associated with HCC occurrence, with BA accumulation often accelerating HCC progression [12,35,36]. Further studies have indicated that various BA species can influence HCC development by modulating cell proliferation, invasion and migration, epithelial-mesenchymal transition (EMT), and angiogenesis within tissue lesions [37,38,39,40,41,42]. Furthermore, investigations have delved into the interplay between BAs and gut microbiota, inflammation, and immunity in the context of HCC progression, shedding light on the mechanisms underlying HCC tumorigenesis.

### 3.1. The Association of Different Species of BAs with HCC 

Alterations in BA profile have been linked to HCC occurrence, with elevated levels of BAs often serving as a risk factor for the development of HCC [12,35]. However, the impact of different BA species on HCC growth varies. A prospective cohort study conducted in Singapore demonstrated that increased levels of conjugated PBAs, primarily taurine-conjugated CDCA, and a reduced ratio of SBA to PBA were associated with a higher risk of HCC incidence, indicating the potential of conjugated PBAs to promote HCC [36]. In parallel, another study revealed that the levels of SBA, especially conjugated DCA, were significantly lower in patients with HCC and DEN-induced HCC mice. Interestingly, the administration of glycol-conjugated DCA effectively suppressed HCC growth in vivo, underscoring the anti-HCC properties of SBAs [41]. Similar findings were observed in NASH-associated HCC patients and mouse models, where the overexpression of steroidogenic acute regulatory protein 1 (STARD1), a transmembrane protein involved in cholesterol transport to mitochondria, led to a significant increase in conjugated PBAs, particularly their tauroconjugates, resulting in enhanced liver tumor multiplicity [7]. Collectively, these findings highlight the contrasting roles of difference BA species, particularly PBAs versus SBAs, in the regulation of HCC growth.

### 3.2. The Regulatory Effect of Various Species of BAs on Tumor Cells

Different BA species have been shown to play distinct roles in HCC growth by regulating intracellular signaling pathways [43]. Specifically, in an in vitro study, DCA, an SBA species, was found to induce endoplasmic-reticulum (ER) stress-coupled JNK signaling, leading to the activation of the intrinsic apoptotic pathway and subsequent suppression of HCC cell growth [44]. However, the inhibitory effect was observed to be nullified in NTCP-negative HCC cells, with DCA treatment instead activating NF-κB/COX-2/IL-8 signaling to promote cell invasion and migration, demonstrating a dual tumor-regulating effect of DCA depending on the cellular context [42]. On the other hand, UDCA, another SBA species, exhibited cytoprotective activities in hepatocytes and inhibited tumorigenesis by inducing cell cycle arrest and apoptosis [45]. Further investigations revealed that UDCA inhibited cell proliferation and induced apoptosis through reactive oxygen species-dependent activation of ERK and dephosphorylation of STAT3, synergizing with sorafenib to enhance its inhibitory efficacy on HCC [46]. Conversely, CDCA, a PBA species, was found to induce actin polymerization and N-cadherin expression, leading to cell malignant transformation via the EMT [33]. CDCA was also shown to repress E-cadherin by inducing the transcription factor Snail, thereby increasing the invasiveness of HCC cells [40]. These findings collectively underscore the pivotal role of BAs in promoting HCC occurrence through the regulation of cell proliferation, invasion and migration, EMT, and apoptosis. Importantly, the distinct effects of different BA species, particularly the disparity between PBAs and SBAs, on HCC growth are mediated through the modulation of multiple signaling pathways. For a comprehensive overview of the regulatory functions of various BA species, refer to Table 1 and Table 2.

### 3.3. The Role of BA Receptors in HCC Growth 

As BAs are characterized by endogenous ligands, their regulation of physiological and pathological processes relies on the mediation of their receptors. Farnesoid X receptor (FXR), a member of the nuclear hormone receptor superfamily, serves as a key BAs receptor involved in modulating the biological effects of BAs [55]. Studies on HCC patients with abnormal BA synthesis have revealed a downregulation of FXR expression, establishing a negative correlation between FXR levels and HCC progression [56]. Notably, FXR knockdown in mice has been shown to trigger spontaneous HCC occurrence during aging, mimicking the pro-tumor effect of FXR downregulation in human [57]. Further investigations have demonstrated that FXR acts as a tumor suppressor by inhibiting HCC growth through the disruption of multiple oncogenic signaling pathways, including NF-κB, STAT3, and wnt/β-catenin pathways [58,59,60], or by functioning as a transcription factor that upregulates the expression of p21, a tumor suppressor, thereby impeding HCC cell proliferation [61]. Additionally, FXR plays a crucial role in maintaining BA homeostasis through negative feedback mechanisms. Loss of FXR leads to BA accumulation, particularly to elevated levels of taurocholic acid (TCA), the conjugated species of PBAs, which in turn induces the expression of Myc, contributing to HCC progression [62].

G-protein-coupled bile acid receptor (TGR5), a member of the membrane-bound G protein-coupled receptors family, is expressed in various tissues and organs, regulating BA homeostasis and energy metabolisms [63]. While direct evidence linking TGR5 levels to HCC progression is lacking, clinical serum samples analysis suggests that hypermethylation of the *TGR5* promoter could serve as a potential biomarker to diagnose HBV-associated HCC. This implies that inhibiting TGR5 expression significantly enhances HCC tumorigenesis [64]. In both in vitro and in vivo experiments, the knockdown of TGR5 in mice heightened sensitivity to DEN-induced liver injury and carcinogenesis. Conversely, overexpression of TGR5 in HepG2 human HCC cells hindered cell proliferation and migration, preliminarily identifying TGR5 as an HCC suppressor [65]. Mechanistic investigations further revealed that TGR5 activation suppresses STAT3 phosphorylation and transcriptional activity, leading to reduced levels of proinflammatory cytokines like IL-6, MCP-1, and IL-10, thereby blocking the development and migration of HCC [65]. Collectively, these findings underscore the pivotal role of BA receptors in the pathogenesis of BA-related HCC.

### 3.4. The Effect of BA–Gut Microbiota Axis on HCC Development

In addition to BA receptors, gut microbiota plays a critical role in modulating BA composition, particularly the conversion from PBAs to SBAs. Different BA species, in turn, affect the community structure and function of gut microbiota. The bidirectional interplay between BAs and gut microbiota has been shown to be associated with HCC progression. An analysis of patients with HCC revealed a significant decrease in the relative abundance of bacterial genera such as *Bifidobacteriales, Lactobacillales, Bacteroidales,* and *Clostridiales,* which are known to possess bacterial bile salt hydrolase (BSH) that hydrolyzes PBAs. This decrease was accompanied by a reduction in the percentage of serum SBAs [41]. In a DEN-induced HCC mouse model, the reduction of BSH-rich bacteria due to vancomycin treatment led to a decrease in DCA level and promoted HCC growth. Notably, the administration of conjugated DCA was able to counteract tumorigenesis, highlighting the significant role of gut microbiota in HCC growth through the regulation of BA profiles [41]. However, a study on obesity-associated HCC demonstrated that a high-fat diet (HFD) stimulated gut bacteria, such as *Clostridium* stains, to increase DCA levels, leading to DNA damage via the production of reactive oxygen species (ROS) and enhancing liver carcinogenesis in mice treated with the chemical carcinogen DMBA [11,66]. The contradictory effect of DCA and related gut bacteria on HCC growth can be attributed to differences in other risk factors in the carcinogenic process. The complexity of these results suggests that the role of BAs is influenced by various contextual factors. Further exploration of BA functions in HCC is warranted across different contexts to gain a comprehensive understanding of their impact.

The effect of BAs on gut microbiota involves decreasing the integrity and increasing the permeability of bacterial cell membrane due to their high hydrophobicity-associated membrane toxicity, ultimately leading to cell death [67]. However, it is important to note that different bacteria display varying sensitivity to the antimicrobial activity of BAs. In vivo experiments revealed that a CA diet resulted in an increase in conditional pathogenic bacteria, such as *Prevotella* and *Desulfovibrio,* while decreasing the levels of beneficial bacteria like *Ruminococcus*, *Lactobacillus*, and *Roseburia*. This shift in gut microbiota composition has been linked to HCC development [68]. The dysfunction of gut microbiota not only impairs the integrity of the intestinal barrier but also upregulates the activities of inhibitory immune cells, such as regulatory T cells (Tregs), which release pro-tumor cytokines like IL-10 and TGF-β, thereby enhancing HCC tumorigenicity [69,70]. These findings underscore the crucial role of the intricate interplay between BAs and gut microbiota in the initiation of HCC.

### 3.5. The Link between BAs and Inflammation in HCC Development

BA dysregulation is a validated etiology associated with HCC, contributing to chronic inflammation through the regulation of signaling pathways that release various inflammatory cytokines. In experiments with DEN-induced HCC mice, it was observed that feeding CAs activated NF-κB signaling, leading to the release of proinflammatory cytokines TNF-α and IL-1β. This, in turn, increased the expression of stemness factors like Myc and Nanog, thereby exacerbating the tumorigenesis and aggressiveness of HCC [71]. A similar outcome was demonstrated in studies involving DCA treatment, where the stimulation of NF-κB signaling triggered the release of TNF-α and IL-6, followed by activation of Janus kinase-signal transducer and activator of the transcription-3 (JAK-STAT3) pathway, consequently elevating the incidence of HCC [72]. Moreover, in cases of dietary or genetic obesity, DCA was found to enhance cellular senescence in hepatic stellate cells (HSCs), leading to the release of inflammatory and tumor-promoting cytokines IL-6 and IL-1β, thereby promoting the development of HCC [11,66]. Other BAs, such as conjugated CDCA, were shown to induce endoplasmic reticulum stress (ERS), resulting in the production of ROS that activate NF-κB, causing DNA damage, liver injury, and HCC progression [73,74,75,76]. In contrast, taurine-conjugated UDCA was found to mitigate ERS-induced inflammatory liver injury by reducing the phosphorylation of eukaryotic initiation factor 2α (elf2α) and the expression of C/EBP homologous protein, thereby slowing down the progression of HCC [77]. Altogether, accumulated BAs act as the mediator that stimulates inflammation, further promoting HCC development.

### 3.6. The Effect of BAs on Immune Response to HCC

Besides the stimulation of inflammation, BAs are able to contribute to the establishment of an immunosuppressive tumor microenvironment (TME) through the modulation of immune cell distribution and activation, which facilitates HCC occurrence and progression. Studies have shown that the regulation of BAs can impact the immune response and promote HCC development. For instance, an increase in conjugated BAs has been linked to the polarization of M2-like macrophages, leading to the creation of an immunosuppressive TME that supports the immune escape of hepatoma cells. In patients with HCC, elevated serum levels of TCA have been associated with the presence of M2-like tumor-associated macrophages (TAMs), while the administration of BA sequestrant cholestyramine has been shown to reversed this effect, inhibiting the promotion of M2-like polarized TAMs and liver tumor growth [5]. Furthermore, the accumulation of SBAs has been found to impede the infiltration of natural killer T (NKT) cells into tumor lesion by suppressing the expression of CXCL16, the solo ligand of CXCR6 expressed on NKT cells, by liver sinusoidal endothelial cells (LSECs). In mouse models, inhibiting the conversion of PBAs to SBAs resulted in increased NKT cell accumulation and reduced liver tumor growth, an effect that could be reversed by administrating SBAs [8,9]. Notably, in nontumor liver tissue from patients with primary liver cancer, a positive correlation was observed between the level of CDCA, a PBA species, and CXCL16 expression, while an inverse correlation was noted between CDCA and the SBA glycolithocholate (GLCA), underscoring the role of BAs in modulating the immune response to HCC [9]. 

Several mechanisms of BAs to induce HCC have been discussed; however, the pathogenesis of tumorigenesis is the comprehensive effect that these elements are orchestrated around BAs. BA accumulation activates NF-κB signaling, promoting HCC cell proliferation and migration, leading to increased expression of proinflammatory cytokine TNF-α and IL-1β [71,72]. TNF-α and IL-1β cause inflammation in tumor tissue and reduce transcription of FXR target genes [78]. Additionally, FXR serves as a negative regulator of hepatic inflammation, with reciprocal suppression observed between FXR and NF-κB signaling pathways, indicating the connection of BA receptors and inflammation in HCC development [59,79]. Similarly, the interaction between BAs and gut microbiota results in the release of inflammatory factors, attracting suppressive immune cells to the tumor lesion, thereby facilitating immune evasion of HCC [5,8,9]. Therefore, understanding the complete mechanism of BAs in HCC development provides rigorous basis for effective therapeutic strategies for HCC.

## 4. Prospects of BA-Based Therapeutics for HCC

The intricate regulatory connection of BAs with their receptors, gut microbiota, inflammation, and immune system provides new approaches for therapeutic inventions against HCC. Accumulated studies have suggested that targeting one of these elements may affect others and result in an additive effect, feasibly preventing HCC development, inhibiting tumor progression, and improving patient prognosis.

### 4.1. BA Receptor Agonists

BA receptor agonists play a crucial role in mediating the function of BAs in cancer. Several agents targeting these receptors have been investigated on their therapeutic efficacy in HCC. FXR, a BA receptor, has been shown to downregulate BA synthesis and counteract their pathological effect [80,81]. Obeticholic acid (OCA), a semi-synthetic BA analogue, acts as a potent agonist to activate FXR [81]. While the effect of OCA on suppressing HCC has been confirmed in mouse models [51], clinical results are still pending. However, several clinical trials are underway to evaluate its therapeutic efficacy in treating BA-related chronic liver disease. Other synthetic FXR agonists, such as MET409, vonafexor, and tropifexor, have shown promising effects in decreasing liver fat content and ameliorating BA-related liver disease in clinical trial [82,83,84,85]. These findings suggest that FXR agonists may serve as alternative therapeutic strategies for treating HCC.

### 4.2. Modulation of BA Metabolism

Modulation of BA metabolism is crucial for maintaining liver homeostasis and has been implicated in HCC development. In this sense, restoration of BA metabolism may offer a potential avenue to slow down HCC progression [62]. BSEP is the BA efflux transporter, and its malfunction results in BA accumulation in the liver [72,86]. FXR is the transcription factor of *BSEP* gene, which promotes its expression by binding to its promoter [72]. Thus, FXR activation induces biliary BA secretion by upregulating the expression of BSEP, preventing BA accumulation in hepatocytes, and effectively retarding HCC progression. BA sequestrants, such as colesevelam and colestipol, are able to chelate BAs to form a non-absorbable complex in the gastrointestinal tract, thus accelerating BA excretion in feces and reducing the enterohepatic circulation of BAs [87]. They were also used to treat HCC in mice models, and the efficacy proved to be beneficial for tumor regression [5]. In addition, these agents also modulate the composition of gut microbiota, inhibiting the proliferation of pathogenic bacteria and blocking HCC development [88]. These data indicate that the modulation of BA metabolism is an alternative therapeutic approach for HCC. 

### 4.3. Regulation of Gut Microbiota

Since BA metabolic disorder caused by dysregulation of gut microbiota is one of the incentives that induce HCC progression, improvement of gut microbiota displays a potential efficacy on the treatment of HCC. In mouse models, administration of probiotics such as *lactobacillus rhamnosus GG* has been shown to inhibit hepatic BA synthesis and enhance BA excretion, leading to the reduction of HCC growth [89]. Similarly, *Lactobacillus brevis* has been found to alleviate the development of HCC by modulating the interaction between gut microbiota and BAs and the activity of Notch 1 signaling pathway [90]. *Lactobacillus eosinophil* can upregulate the expression of FXR and FGF15, improving NAFLD symptom in mouse models [91]. Importantly, the effect of probiotic mixtures such as various *Lactobacillus* strains on the BA profile has been assessed in clinical trials, and beneficial action was preliminarily exhibited [92]. In addition, prebiotics that stimulate the growth and activity of beneficial bacteria have been used to alleviate metabolic disorder of BAs, and dietary interventions tailored to the gut microbiota are also attempted to prevent chronic liver disease and HCC [93,94]. These findings highlight the potent approach of modulating gut microbiota for preventing HCC growth. 

### 4.4. Combination Therapy 

Since the intricate connection of BAs with their receptors, gut microbiota, and the immune response orchestrates to induce HCC progression, combination therapy targeting these elements has been shown to be more efficient in the treatment of HCC. UDCA has been demonstrated to restrain Treg cell differentiation and activation by degrading TGF-β. Additionally, UDCA synergizes with anti-PD-1 to enhance antitumor immunity and tumor-specific immune memory in an HCC mouse model [15]. In line with the result, retrospective analysis has indicated that combining UDCA with atezolizumab (anti-PD1) or bevacizumab (anti-VEGF) is more effective in patients with HCC compared to monotherapy [14,15]. Furthermore, the combination of UDCA with sorafenib chemotherapy has shown a synergistic effect on anti-HCC activity by regulating STAT3 and ERK signaling pathways [46]. Moreover, combining chemotherapy and radiotherapy with UDCA or TUDCA not only enhances HCC therapeutic efficacy, but also mitigates side effects by modulating BA levels [77,95,96]. Ongoing experiments on combination therapy hold promise for developing more effective strategies for HCC therapy.

In summary, the involvement of BA metabolic disorder in HCC progression has been confirmed, highlighting the potential of restoring BA metabolism as an effective avenue to suppress liver tumor growth. Various approaches targeting BA receptors, gut microbiota, and the immune response have been suggested and tested in animal models and clinical practices, given the multiple factors regulating BA synthesis, functions, and interactions. Importantly, combination therapy involving multiple agents has shown greater efficacy in treating HCC compared to monotherapy.

## 5. Conclusions and Outlook

The disturbance of BA metabolism is closely related to liver tumorigenesis. Accumulated BAs induce multiple pathological processes, such as tumor cell proliferation, invasion and metastasis, EMT regulation, angiogenesis, and apoptosis, through the regulation of various signaling pathways mediated by BA receptors. The intricate interplay of BAs with gut microbiota, inflammation, and immune response has provided new evidence to understand the complex pathogenesis of this malignancy. Elucidation of the crosstalk among BA, gut microbiota, and immune response to HCC contributes to the exploration of innovative therapeutic approaches. Based on these findings, the modulation of BA receptors, gut microbiota, and BA profile is confirmed to be a novel approach to treating HCC, and maintenance of BA homeostasis is critical to prevent HCC development. 

Several limitations warrant consideration in the development of safe and effective therapeutic strategies targeting BAs and gut microbiota for HCC prevention and treatment. Challenges such as translating experimental results to clinical application highlight the need for urgent clinical trials to verify the efficacy of these strategies. It is crucial to address the diverse functions of different BA species in HCC progression, as well as the conflicting findings regarding the roles of specific BAs in various contexts. Further research is needed to elucidate the specific role of BAs in different scenarios. Additionally, a comprehensive understanding of the metabolic kinetics, pharmacodynamics, and potential side effects of BA-related agents is essential for evaluating their antitumor effects thoroughly. Moreover, the individualized nature of gut microbiota underscores the importance of considering personal characteristics in the development of therapeutic strategies targeting BA metabolism and bacterial composition. 

In conclusion, an exciting field of research lies in the association of BA metabolism with HCC progression, where the interaction between BAs and their receptors, gut microbiota, and immune response plays a crucial role in the development of HCC. Novel therapeutic avenues aimed at targeting these elements have been discovered to retard HCC progression, ultimately enhancing the prognosis and quality of life for patients with HCC. Continued studies on the interaction between these components are anticipated to yield valuable evidence that will guide the development of innovative therapies for this malignant disease. 

## Figures and Tables

**Table 1:** Tumor-suppressive role of BAs in HCC.

Bile Acids	Cell Line	Effects	Ref
DCA	Huh-BAT, Huh-7, SNU-761, SNU-475	Induction of apoptosis in NTCP-positive HCC cells, while inhibition of invasion of NTCP-negative HCC cells via NF-κB/COX-2/IL-8 signaling in combination with cyclooxygenase inhibitors	[42]
UDCA	Huh-BAT, HepG2	Inhibition of proliferation and induction of apoptosis via activation of ERK and dephosphorylation of STAT3 in synergy with sorafenib	[46]
	HepG2, BEL7402	Inhibition of proliferation and induction of apoptosis via blocking cell cycle and regulating the expression of Bax/Bcl-2 genes	[47]
	HepG2, SK-Hep1, SNU-423, Hep3B	Switch of oxaliplatin-induced necrosis to apoptosis via inhibiting of ROS production and activating of the p53-caspase 8	[48]
	Huh-BAT, SNU-761, SNU-475	Suppression of cell growth and induction of DLC1 tumor suppressor protein expression	[49]
	HepG2	Induction of apoptosis via regulating the ratio of Bax/Bcl-2 and the expressions of Smac, Livin, and caspase-3	[50]
	Huh 7	Iinhibition of angiogenesis through suppressing HIF-1α/VEGF/IL-8	[45]
Obeticholic acid (OCA)	HepG2, Huh7, SNU-449	Inhibition of proliferation, metastasis, and invasion through interfering IL-6/STAT3	[51]
GDCA	SUN-449, HepG2	Inhibition of proliferation and migration, induction of apoptosis	[41]

**Table 2:** Tumor-promotive role of BAs in HCC.

Bile Acids	Cell Line	Effects	Ref
CDCA	Huh-7, Hep3B	Enhancement of the TGF-β-induced EMT	[39]
CDCA	Hep3B	Induction of Snail expression and downregulation of E-cadherin expression to induce migration and invasion	[40]
LCA	Hep3B	Induction of Snail expression and downregulation of E-cadherin expression to induce migration and invasion	[40]
Norcholic Acid (NorCA)	LM3, Huh-7	Promotion of proliferation, migration, and invasion by negatively regulating FXR	[14]
OCA	HepG2	Induction of proliferation via stimulating MAFG expression	[52]
GCDCA	HepG2	Enhancement of antiapoptotic and proliferation by activating ERK1/2 inducing Mcl-1 phosphorylation at T163 to stabilize Mcl-1 protein	[53]
	SMMC-7721, Huh7	Promotion of invasion via activating autophagy by targeting the AMPK/mTOR pathway	[54]

## Data Availability

Not applicable.
